# Pu-erh Tea Regulates Fatty Acid Metabolism in Mice Under High-Fat Diet

**DOI:** 10.3389/fphar.2019.00063

**Published:** 2019-02-05

**Authors:** Fengjie Huang, Shouli Wang, Aihua Zhao, Xiaojiao Zheng, Yunjing Zhang, Sha Lei, Kun Ge, Chun Qu, Qing Zhao, Chao Yan, Wei Jia

**Affiliations:** ^1^School of Pharmacy, Shanghai Jiao Tong University, Shanghai, China; ^2^Shanghai Key Laboratory of Diabetes Mellitus and Center for Translational Medicine, Shanghai Jiao Tong University Affiliated Sixth People’s Hospital, Shanghai, China; ^3^University of Hawaii Cancer Center, Honolulu, HI, United States

**Keywords:** Pu-erh tea, free fatty acids, lipid lowering effect, fatty acid β-oxidation, obesity

## Abstract

Pu-erh tea has been extensively reported to possess lipid lowering effects but the underlying mechanisms remained unclear. Free fatty acids (FFAs) are generally correlated with the development of obesity, leading to increased risk for type 2 diabetes mellitus and cardiovascular diseases. To investigate whether Pu-erh tea treatment alters FA metabolism, we treated HFD induced obese mice with Pu-erh tea for 22 weeks and analyzed FFA profiles of experimental mice using a UPLC-QTOF-MS platform. Results showed remarkable changes in metabolic phenotypes and FFA compositions in mice treated with or without Pu-erh tea. HFD induced a marked obese phenotype in mice as revealed by significantly increased body weight, liver and adipose tissue weight, lipid levels in serum and liver, and these parameters were markedly reduced by Pu-erh tea treatment. Several FFA or FFA ratios, such as DGLA, palmitoleic acid, and OA/SA ratio, were significantly increased while the levels of SA/PA and AA/DGLA were significantly reduced in HFD-induced obese mice. Interestingly, these differential FFAs or FFA ratios were previous identified as key markers in human obese subjects, and their changes observed in the HFD group were reversed by Pu-erh tea treatment. Moreover, a panel of FFA markers including C20:3 n6/C18:3 n6 and C20:3 n6/C20:2 n6, C18:3 n6/C18:2 n6, C18:3 n3/C18:2 n6 and C24:1 n9/C22:1 n9, which were previously identified as biomarkers in predicting the remission of obesity and diabetes in human subjects who underwent metabolic surgery procedures, were reversed by Pu-erh tea intervention. Pu-erh tea significantly improved glucose homeostasis and insulin tolerance compared to the HFD group. Additionally, Pu-erh tea treatment significantly decreased FFA synthesis genes and increased the expression of genes involved in FFA uptake and β-oxidation including FATP2, FATP5, PPARα, CPT1α, and ACOX-1. These finding confirmed the beneficial effects of Pu-erh tea on regulating lipid and glucose metabolism, and further validated a panel of FFA markers with diagnostic and prognostic value for obesity and diabetes.

## Introduction

Obesity, as a key component of metabolic syndrome, is closely associated with the risk of developing cardiovascular disease, type 2 diabetes and even many types of cancer (Kolotkin et al., 2001; Ogden et al., 2014). There are a number of possible pathophysiological mechanisms involved in the development and maintenance of obesity. Previous studies suggested that the obesity is highly correlated with FFAs which are derived from adipocytes through lipolysis (Boden and Shulman, 2002; Boden, 2011). Recently, our group has reported several plasma FFAs as reliable markers in predicting the future metabolic abnormalities in obese subjects, such as dihomo-gamma-linolenic acid (DGLA), stearic acid/palmitic acid(SA/PA) ratio, oleic acid/stearic acid (OA/SA) and arachidonic acid/dihomo-γ-linolenic acid (AA/DGLA) ratios (Ni et al., 2015; Zhao et al., 2016, 2017).

Pu-erh tea, a fermented black tea made from young leaves of *Camellia sinensis*, is well known for its unique aroma and taste. Accumulating lines of evidence demonstrated that Pu-erh tea can reduce the body weight, suppress hyperlipidemia, lower triglyceride, total cholesterol and low-density lipoprotein-cholesterol levels, and increase high-density lipoprotein cholesterol levels (Chu et al., 2011; Qiong and Xishuang, 2014; Cai et al., 2017). However, the specific effect of Pu-erh tea on FA metabolism is under investigated and we suspect that there is a mechanistic link between the FA metabolism and the anti-obesity effect of Pu-erh tea. Thus, we conducted this study to investigate the anti-obesity and hypolipidemic effect of Pu-erh tea in an obese mouse model induced by high-fat diet (HFD).

There are a variety of Pu-erh tea products produced using different fermentation processes and/or tea leaves collected from different plantation locations. The phytochemical components may vary greatly among different products/batches of Pu-erh tea. In this study we selected an instant Pu-erh tea product manufactured using a well-established, standardized fermentation and extraction process to maintain consistent chemical constituents among different batches of Pu-erh tea and achieve reproducible results. The instant Pu-erh tea used in our study contained most of the components in ripe Pu-erh tea and was more homogeneous in controlling the dosage of the tea infusion, based on our previous evaluation of Pu-erh tea products.

## Materials and Methods

### Chemicals and Reagents

Control diet contained 10% lipids, 19% proteins, and 71% carbohydrates, while the HFD contained 45% lipids, 19% proteins, and 36% carbohydrates (Trophic Animal Feed High-tech Co. Ltd., Nantong, China). The Pu-erh tea infusions were prepared by dissolving 600 mg of the Pu-erh tea powder (a commercial product, Deepure, acquired from Tasly Pharmaceutical Co. Ltd., Tianjin, China) with 200 mL pure sterilized water. A total of 54 FFA standards were obtained from Sigma-Aldrich, TRIzol Reagent (Invitrogen, Life Technology, United States), Prime Script RT Reagent Kit (TAKARA, Kusatsu, Japan), and Power Up SYBR Green PCR Master Mix (Applied Biosystems, Thermo Fisher Scientific, United States). The tea powder were extracted by water and methanol respectively and the dominating compounds in instant Pu-erh tea were analyzed by UPLC-QTOF-MS (Supplementary Figure 1 and Supplementary Table 1). The dominating compounds include L-arginine, Epicatechin gallate, Gallocatechin gallate, Myricetin, Caffeine and Theophylline identified in positive ion mode and Gallic acid, Catechin, Epcatechin, Gallocatechin, Epigallocatechin, Catechin gallate, Kaempferol, Quercetin, Quercetin-3-*O*-Glu and Kaempferol-3-*O*-Glu identified in negative ion mode.

### Animal Study and Sample Collection

This study was carried out in accordance with the recommendations of the national legislation and local guidelines of the Laboratory Animals Center at Shanghai Jiao Tong University Affiliated Sixth People’s Hospital, Shanghai, China. The protocol were reviewed and approved by the Institutional Animal Care and Use Committee at the Center for Laboratory Animals, Shanghai Jiao Tong University Affiliated Sixth People’s Hospital, Shanghai, China.

C57BL/6J mice (male, 3 weeks old) were purchased from Shanghai Laboratory Animal Co. Ltd. (SLAC, Shanghai, China). All the mice were maintained in a specific-pathogen-free (SPF) environment with controlled conditions of a 12 h light/dark cycle at 20–22°C and 45 ± 5% humidity. Mice were acclimated on a control chow diet *ad libitum* for 1 week and then randomly divided into three groups: control group received normal chow (Control Diet), HFD, and HFD with Pu-erh tea infusion group at a dosage of 450 mg/Kg per day (HFD + Pu-erh Tea). The Pu-erh tea infusions were prepared for a concentration of 3 mg/mL by dissolving 600 mg of tea powder with 200 mL pure sterilized water. All the mice were raised with free access to control chow/HFD and water/tea infusion, and their body weights were recorded once a week for 22 weeks. At the end of these experiments, mice were fasted overnight before being euthanized. Blood samples were collected and then kept at room temperature for half an hour before centrifugation at 4°C, 5000 rpm for 10 min to obtain the serum samples. Tissues including liver, abdominal adipose tissue, perirenal fat and subcutaneous fat were carefully collected, kept in liquid nitrogen, and then stored at −80°C until analysis.

### Sample Preparations and FFA Analysis

The FFA in serum and liver tissues were extracted and quantified as previously described (Zheng et al., 2016). Briefly, samples were weighed and extracted with mixture of isopropanol and hexane containing phosphate by homogenization and centrifugation. The supernatant was mixed with internal standard (nonadecylic acid-D37) and subsequently extracted with hexane and water. The mixture was then transferred to a new tube and dried with vacuum and further reconstituted with methanol for instrumental analysis with UPLC-QTOF-MS (Waters Corp., Milford, MA, United States).

The instrumental parameters of the analysis were set as follows. The mobile phase were water (A) and acetonitrile/isopropanol (v/v = 80/20, B). A BEH C18 (2.1 mm × 100 mm, 1.7 μm) chromatographic column was used for compound separation with a flow rate of 0.4 mL/min and column temperature at 40°C. The elution gradient were 70% B (0–2 min), 70–75% B (2–5 min), 75–80% B (5–10 min), 80–90% B (10–13 min), 90–99% B (13–16 min) and kept at 99% B for 5 min before switching back to initial condition. The MS was operated at a negative electrospray ion mode with a capillary voltage of 2.5 kV, sampling cone at 55 V, extraction cone at 4 V, source temperature at 150°C and desolvation temperature at 450°C. A standard calibration solution with 54 FFA standards at 11 different concentration levels were analyzed to construct the calibration curve. Peak annotation and quantitation were conducted by TargetLynx application manager (Waters Corp., Milford, MA, United States).

### Histological Staining of Liver Section

Liver tissues were fixed in 10% neutral-buffered formalin, embedded in paraffin blocks, and processed for routine H&E staining. The stained sections were subsequently examined for histopathological changes.

### Measurement of Biochemical Parameters and Hepatic Lipids

The serum TC, TG, HDL, and LDL were measured using a TBA-40FR Automatic Biochemical Analyzer (TOSHIBA, Japan), according to the manufacturer’s protocol. Hepatic lipids were extracted by the Folch method. Briefly, the liver tissues were homogenized with a chloroform/methanol (2/1, v/v) solution to a final volume 20 times that of the tissue sample and followed by a series of dispersion, agitation, and centrifugation steps. The hepatic total cholesterol (TC) and triglyceride (TG) were measured using Elisa kits (BluGene Biotech, Shanghai, China) according to the manufacturer’s instructions.

### Oral Glucose Tolerance Test (OGTT) and Insulin Tolerance Test (ITT)

OGTT and ITT were performed after 12 and 4 h fasting respectively. About 1 g/Kg of glucose was given by oral gavage for OGTT test and 0.75 units/Kg of insulin were given by intraperitoneal injection. Blood samples at 0, 15, 30, 60, and 120 min were collected from caudal vein. The blood glucose levels were measured and the area under the curve (AUC) during the OGTT and ITT test were calculated.

### Real-Time Quantitative PCR

The liver tissues were homogenized and total RNA was isolated using TRIzol Reagent. The total RNA concentration was measured using a NanoDrop 2000C spectrophotometer (Thermo Fisher Scientific, Waltham, MA, United States). Total RNA was reverse transcribed using random hexamer primers to form the cDNA templates employing a Prime Script RT Reagent Kit. The quantitative real-time PCR reaction mixture was set up using Power Up SYBR Green PCR Master Mix and the reaction was run in an ABI 7900HT Real-Time PCR System (Applied Biosystems Instruments, Thermo Fisher Scientific, United States). All the procedures were performed following the manufacturer’s instructions. GAPDH was used as a house-keeping gene and the relative expression of the target genes was calculated using the comparative Ct approach (dCT), and ultimately the relative expression was used as fold changes relative to the control group.

### Statistical Analysis

Raw data from FFA quantification were obtained with MassLynx v4.1 and analyzed by TargetLynx v4.1 (Waters, Milford, MA, United States). All the bar plots in this study were generated with GraphPad Prism 6.0 (GraphPad Software, San Diego, CA, United States), and differential analysis with Mann–Whitney *U* test was conducted using SPSS 20.0 (IBM SPSS, United States) with significant criteria set to be ^∗^*p*-value < 0.05 and #*p*-value < 0.005.

## Results

### Pu-erh Tea Attenuated HFD Induced Body Weight Gain and Increase of Lipid Levels in Serum and Liver of Mice

**FIGURE 1:**
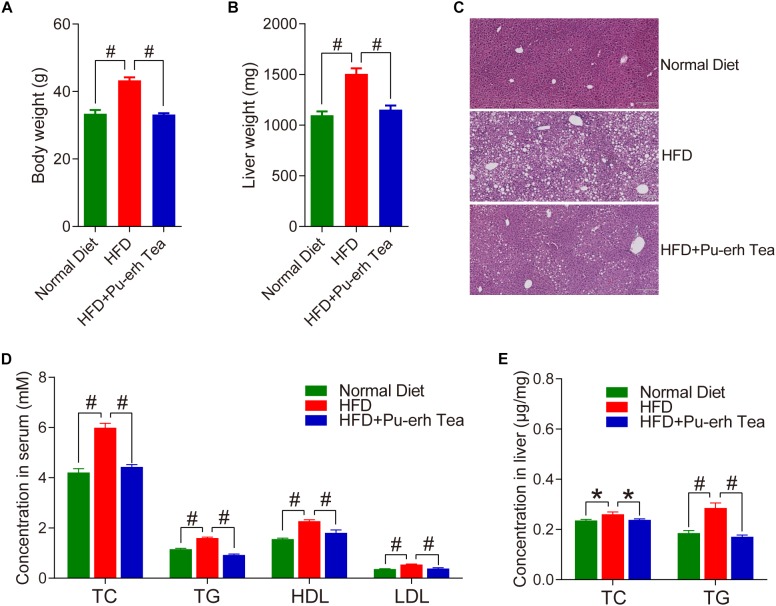
Pu-erh tea significantly reduced HFD-induced increase in body weight and lipids in serum and liver of C57BL/6 mice. **(A)** Body weight of mice fed with control diet, HFD, and HFD with 450 mg/Kg/day of Pu-erh tea across 22 weeks of intervention. **(B)** Liver weight of mice fed with control diet, HFD, and HFD with 450 mg/Kg/day of Pu-erh tea for 22 weeks. **(C)** HE staining of liver section of mice fed with control diet, HFD, and HFD with 450 mg/Kg/day of Pu-erh tea for 22 weeks. **(D)** Concentrations of TC, TG, HDL, and LDL in serum of mice fed with control diet, HFD, and HFD with 450 mg/Kg/day of Pu-erh tea for 22 weeks. **(E)** Concentration of TC and TG in liver of mice fed with control diet, HFD, and HFD with 450 mg/Kg/day of Pu-erh tea for 22 weeks. Data were expressed as mean ± SEM and differences were assessed by Mann–Whitney *U* test, ^∗^*p* < 0.05, #*p* < 0.005.

Male C57BL/6 mice were fed with normal diet (Normal Diet group), high fat diet (HFD group) and HFD coupled with 3 mg/mL instant Pu-erh tea infusion in their water bottles (HFD + Pu-erh Tea group) for 22 weeks. The body weight of mice was significantly increased in HFD group which were robustly attenuated by Pu-erh tea (Figure 1A). The liver weight of mice was significantly increased in HFD group and decreased in Pu-erh tea group (Figure 1B). Meanwhile, HE staining of the liver section showed that the lipid droplets in HFD group were significantly increased and larger than normal control and the Pu-erh tea consumption improved the condition (Figure 1C). These results indicated that the Pu-erh tea reversed the HFD induce obesity and fatty liver in C57BL/6 mice. As the lipid lowering effect is another well known beneficial function of Pu-erh tea, we measured the serum biochemical markers of TC, TG, HDL, LDL and hepatic TC and TG. The results showed that the serum lipids and hepatic lipids were significantly elevated by HFD and were reversed by Pu-erh tea consumption (Figure 1D,E). Taken together, these results revealed that long term HFD intervention induced obesity, fatty liver, and hyperlipidemia in C57BL/6 mice, and these abnormal conditions can be improved by concurrent consumption of Pu-erh tea.

### Pu-erh Tea Reversed HFD-Induced Changes in FFA Profiles

**FIGURE 2:**
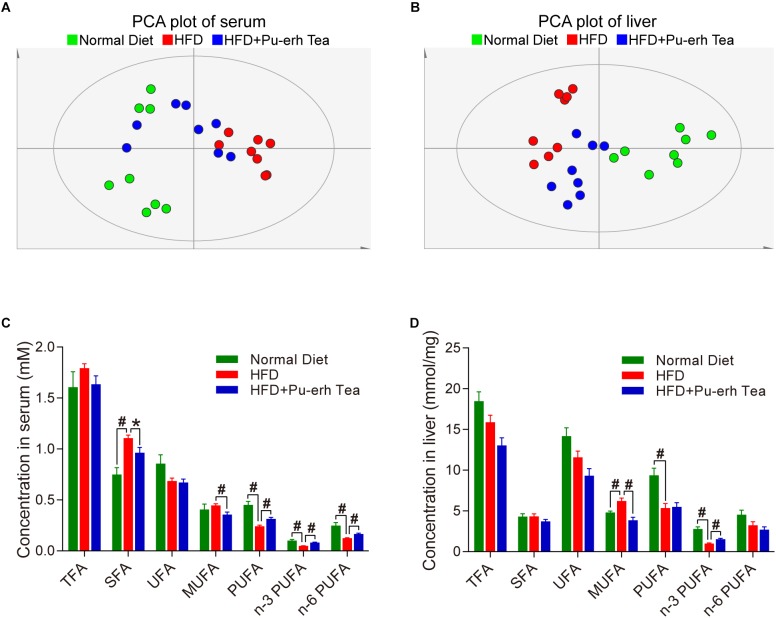
HFD and Pu-erh tea altered FFA profiles and main FFA species in both serum and liver of mice. **(A)** Principle component analysis (PCA) plot based on serum FFA profile of mice fed with control diet, HFD, and HFD with 450 mg/Kg/day Pu-erh tea for 22 weeks. **(B)** PCA plot based on hepatic FFA profile of mice fed with control diet, HFD, and HFD with 450 mg/Kg/day Pu-erh tea for 22 weeks. **(C)** FFA species in serum of mice fed with control diet, HFD, and HFD with 450 mg/Kg/day Pu-erh tea for 22 weeks. **(D)** FFA species in liver of mice fed with control diet, HFD, and HFD with 450 mg/Kg/day Pu-erh tea for 22 weeks. Data were expressed as mean ± SEM and differences were assessed by Mann–Whitney *U* test, ^∗^*p* < 0.05, #*p* < 0.005.

We compared the FFA profiles in normal diet, HFD, and HFD coupled with Pu-erh tea group using a multivariate analysis model. The PCA plot of both serum and liver showed that the samples in HFD group were well separated from those in the Normal Diet group, and the samples in HFD+Pu-erh Tea group changed their spatial position moving toward the Normal Diet group (Figure 2A,B). This indicated that the changes of FFA profile induced by HFD were significantly modulated by Pu-erh tea treatment. The specific changes of different FFA species were further analyzed. In serum, saturated fatty acids (SFA) as well as monounsaturated fatty acids (MUFA) were increased in HFD and such increase was subsequently inhibited by Pu-erh tea consumption. Meanwhile, the polyunsaturated fatty acids (PUFA) including n-3 and n-6 series were decreased in HFD group and elevated in HFD + Pu-erh Tea group (Figure 2C). In the meanwhile, the changes of MUFA and n-3 PUFA in the liver were resemble to those in serum including elevated MUFA and attenuated n-3 PUFA levels in HFD group and subsequently normalized in HFD + Pu-erh Tea group (Figure 2D).

### Differential FFAs Altered by HFD and Pu-erh Tea

**FIGURE 3:**
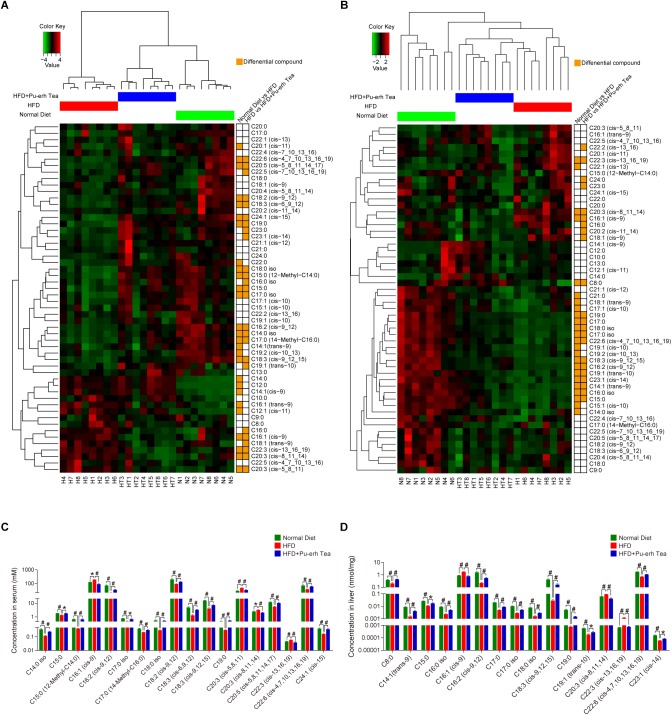
FFA profile and differential FFAs induced by HFD and Pu-erh tea treatment. **(A)** Heatmap based on *Z*-scores of serum FFAs in mice fed with control diet, HFD, and HFD with 450 mg/Kg/day Pu-erh tea for 22 weeks. **(B)** Heatmap based on *Z*-scores of hepatic FFA profile of mice fed with control diet, HFD, and HFD with 450 mg/Kg/day Pu-erh tea for 22 weeks. **(C)** Concentrations of differential FFAs in serum of mice fed with control diet, HFD, and HFD with 450 mg/Kg/day Pu-erh tea for 22 weeks. **(D)** Concentrations of differential FFA in liver of mice fed with control diet, HFD, and HFD with 450 mg/Kg/day Pu-erh tea for 22 weeks. Data were expressed as mean ± SEM and differences were assessed by Mann–Whitney *U* test, ^∗^*p* < 0.05, #*p* < 0.005.

The differential FFAs were identified based on the following criteria (Supplementary Table 2): (1) *P*-value of Mann–Whitney *U* test between tested two groups were less than 0.05; (2) fold change between two groups were greater than 1.2 or less than 0.8; (3) variable importance in projection (VIP) values of the orthogonal partial least square-discriminate analysis (OPLS-DA) between tested two groups were greater than 1.0. The OPLS-DA plot of FFAs in serum and liver showed better separation than PCA plot. The *Z*-scores of FFA in all samples were calculated for hierarchical clustering of samples and compound dimensions and further visualized using a heatmap chart. As a result, the samples in three groups were well clustered to each group and the HFD + Pu-erh Tea group lies between the Normal Diet group and HFD group (Figure 3A,B). Most of the differential FFAs were clustered as the same variants in both HFD and HFD + Pu-erh Tea groups. The absolute concentration of differential FFAs in serum and liver showed SFAs were decreased in HFD and increased in HFD + Pu-erh Tea group, and part of UFA showed same trend while others were increased in the HFD group and further attenuated by Pu-erh tea treatment (Figure 3C,D). These differential FFAs were potential biomarkers and may be involved in altered enzymatic activities in FA metabolism. Further investigation of the identified FFA markers related to significantly altered activity of metabolic enzymes may help identify novel therapeutic targets for the treatment of obesity in the future.

### Pu-erh Tea Regulated FA Metabolism in Obesity

**FIGURE 4:**
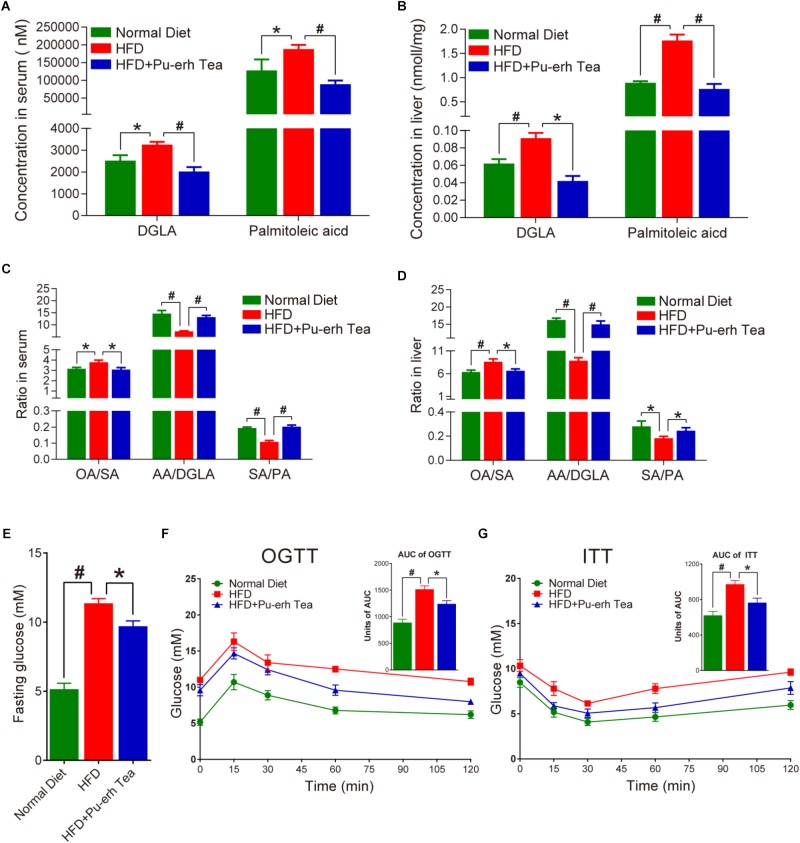
Pu-erh tea altered FFA biomarker of obesity. **(A)** Concentrations of DGLA and palmitoleic acid in serum of mice fed with control diet, HFD, and HFD with 450 mg/Kg/day Pu-erh tea for 22 weeks. **(B)** Concentrations of DGLA and palmitoleic acid in liver of mice fed with control diet, HFD, and HFD with 450 mg/Kg/day Pu-erh tea for 22 weeks. **(C)** Ratios of OA/SA, AA/DGLA, and SA/PA in serum of mice fed with control diet, HFD, and HFD with 450 mg/Kg/day Pu-erh tea for 22 weeks. **(D)** Ratios of OA/SA, AA/DGLA, and SA/PA in liver of mice fed with control diet, HFD, and HFD with 450 mg/Kg/day Pu-erh tea for 22 weeks. **(E)** Fasting glucose level in serum of mice fed with control diet, HFD, and HFD with 450 mg/Kg/day Pu-erh tea for 22 weeks. **(F)** Oral glucose tolerance test (OGTT) and the related area under the curve (AUC) of mice fed with control diet, HFD, and HFD with 450 mg/Kg/day Pu-erh tea at the end of week 18. **(G)** Insulin tolerance test (ITT) and the related area under the curve (AUC) of mice fed with control diet, HFG, and HFD with 450 mg/Kg/day Pu-erh tea at the end of week 19. Data were expressed as mean ± SEM and differences were assessed by Mann–Whitney *U* test, ^∗^*p* < 0.05, #*p* < 0.005.

Mice fed HFD showed significantly increased levels of DGLA and palmitoleic acid which were normalized by Pu-erh tea treatment in both serum and liver (Figure 4A,B). Other FFAs including GLA (C18:3 n6), PA (C16:1 n7), HA (C17:1 n7) were also markedly altered. HFD-induced obese mice showed higher level of OA/SA and lower level of AA/DGLA and SA/PA, whereas Pu-erh tea consumption attenuated all of these changes (Figure 4C,D). Moreover, HFD mice showed elevated levels of fasting glucose and Pu-erh tea treatment significantly improved fasting glucose compared to HFD mice (Figure 4E). We further performed OGTT and ITT tests to determine the impact of Pu-erh tea treatment on glucose homeostasis and insulin sensitivity. Results showed that long-term HFD induced glucose intolerance which was significantly improved by Pu-erh tea treatment (Figure 4F). Similarly, insulin sensitivity of mice was impaired in HFD group and became significantly improved in HFD + Pu-erh Tea group (Figure 4G).

**FIGURE 5:**
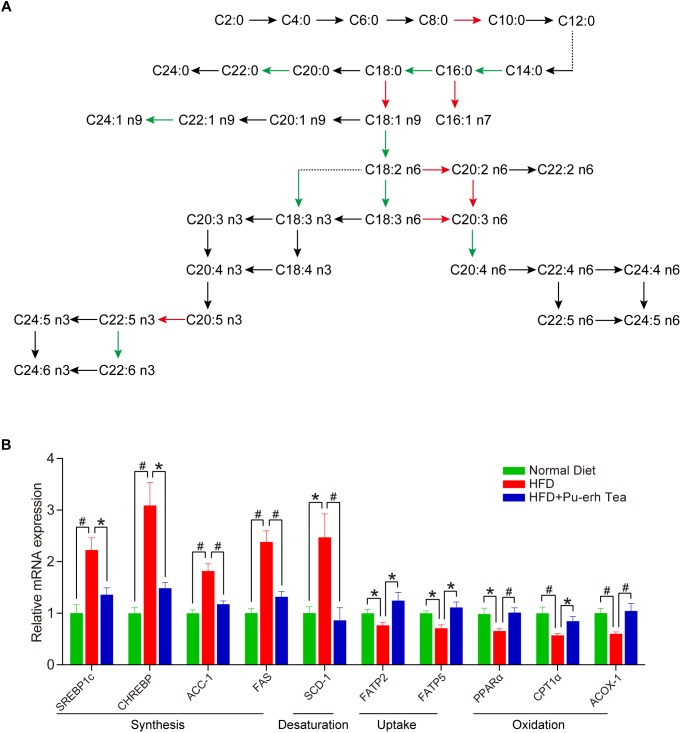
Pu-erh tea altered FA related enzymatic activities and gene expression. **(A)** Biochemical synthesis pathway related to FA metabolism and ratios regulated by HFD and Pu-erh tea. Red arrows represent the ratios significantly elevated by HFD but attenuated by Pu-erh tea, while the green arrows represent the ratios significantly lowered by HFD but elevated by Pu-erh tea. **(B)** Hepatic gene expression related to FA metabolism of mice fed with control diet, HFD, and HFD with 450 mg/Kg/day Pu-erh tea for 22 weeks. Data were expressed as mean ± SEM and differences were assessed by Mann–Whitney *U* test, ^∗^*p* < 0.05, #*p* < 0.005.

To further investigate whether metabolic enzymes involved in the FA biosynthesis pathway were altered in response to HFD and Pu-erh tea interventions, a total of 30 FA product/precursor ratios which represent the elongase and desaturase enzyme activities in FA synthesis were calculated. As a result, 8 elongase ratios including C10:0/C8:0, C16:0:C14:0, C18:0/16:0, C22:0/C20:0, C24:1 n9/C22:1 n9, C20:2 n6/C18:2 n6, C20:3 n6/C18:3 n6, C22:5 n3/C20:5 n3 and 8 desaturase ratios including C16:1 n7/C16:0, C18:1 n9/C18:0, C18:2 n6/C18:1 n9, C18:3 n6/C18:2 n6, C18:3 n3/C18:2 n6, C20:3 n6/C20:2 n6, C20:4 n6/C20:3 n6, and C22:6 n3/C22:5 n3 were significantly altered in serum or liver in HFD group and HFD + Pu-erh Tea group, respectively (Figure 5A). Among these FA product/precursor ratios, 8 ratios including C18:0/C16:0 (SA/PA), C18:1 n9/C18:0 (OA/SA), C18:3 n6/C18:2 n6 (GLA/LA), C18:3 n3/C18:2 n6, C20:2 n6/C18:2 n6, C20:3 n6/C18:3 n6 (DGLA/GLA), C20:4 n6/C20:3 n6 (AA/DGLA), and C22:5 n3/C20:5 n3 (DPA/EPA) were significantly altered in both serum and liver in same trend (Figure 5A). The ratios of C20:3 n6/C18:3 n6 (DGLA/GLA) and C20:3 n6/C20:2 n6 were significantly increased in HFD mice and decreased in HFD + Pu-erh Tea group while the ratio of C18:3 n6/C18:2 n6 (GLA/LA), C18:3 n3/C18:2 n6 and C24:1 n9/C22:1 n9 were decreased in HFD mice and increased in HFD + Pu-erh Tea group (Figure 5A).

We further quantitatively measured the mRNA expression levels of genes involved in FA synthesis, desaturation, uptake and oxidation in the liver. The expression of SREBP1c, CHREBP and ACC-1 was significantly increased in the HFD group and CHREBP were decreased in HFD + Pu-erh tea group (Figure 5B). SCD-1, a key rate limiting enzyme in the desaturation of FFA, was significantly elevated by HFD and attenuated in the HFD + Pu-erh tea group (Figure 5B). The changes of SCD-1 were relevant to the changes in C18:1 n9/C18:0 (OA/SA) and C16:1 n7/C16:0 ratios. Moreover, the genes related to hepatic FA uptake showed that the expression of CD36 was highly activated in HFD group but inhibited in Pu-erh tea group, while the expression of FATP2 and FATP5 was significantly reduced in HFD group but activated by Pu-erh tea treatment (Figure 5B). PPARα, CPT1α, ACOX-1 were concurrently decreased in HFD group but significantly increased in HFD + Pu-erh tea group (Figure 5B). These results indicated that Pu-erh tea treatment reduced FA synthesis, desaturation and promoted FA uptake and β-oxidation which may be essential factors in regulating FA metabolism.

## Discussion

Our results showed that Pu-erh tea significantly reduced body weight, liver weight, and adipose tissue weight along with decreased levels of serum lipids including TC, TG, HDL, LDL and hepatic lipids of TC and TG. These results further confirmed the weight loss and lipid-lowering effects of Pu-erh tea that have been previously reported.

Dysregulated FA metabolism is closely associated with altered FA and lipid profiles and mechanistically involved in the development of obesity and diabetes. In our previous study, the concentrations of DGLA and palmitoleic acid in serum were significantly increased in obese subjects compared to their healthy counterparts, and thus, their levels could predict the recurrence of diabetes in obese subjects with diabetes who underwent metabolic surgery (Ni et al., 2015). In this study, mice fed with HFD showed increased level of DGLA and palmitoleic acids which can be readily suppressed by Pu-erh tea consumption. Moreover, enzymatic activities involved in FA metabolism (biosynthesis, desaturation, uptake, and β oxidation), as identified by FA product to precursor rations as well as their expression levels were significantly altered by HFD intervention and subsequently attenuated or normalized by Pu-erh tea treatment. Previous studies uncovered that among potential product/precursor FFA ratios, OA/SA was significantly elevated and AA/DGLA, SA/PA were significantly decreased in the progression from metabolic healthy obese (MHO) to metabolic unhealthy obese (MUO) (Zhao et al., 2016, 2017). Moreover, multivariate logistic regression showed that the baseline level of OA/SA was a positive predictor, and AA/DGLA, SA/PA were negative predictors for the development of MUO from MHO (Zhao et al., 2016). Another interventional study analyzed 38 obese subjects who received very low carbohydrate diet (VLCD) dietary intervention, which showed that the ratio of OA/SA was significantly decreased whereas ratios of AA/DGLA and SA/PA were significantly elevated together with significant reduction of BMI, subcutaneous and visceral fat compared with pre-dietary treatment baseline (Zhao et al., 2016). These studies also investigated the elongase and desaturase activities of obese subjects with T2DM remission after RYGB surgery. The result revealed that higher baseline level of SA/PA ratio, reflecting an elovl6-encoded elongase enzyme activity, was associated with greater probability of diabetes remission after RYGB and may serve as a diagnostic marker in preoperative patient assessment. In this study, long term HFD also induced higher level of OA/SA and lower level of AA/DGLA, and SA/PA. These changes can be reversed by Pu-erh tea consumption. Our study further showed that 22 weeks of HFD intervention elevated fasting glucose levels and affected glucose tolerance and insulin tolerance level. These metabolic abnormalities were markedly improved in HFD + Pu-erh tea group.

A number of studies investigated the gene expression relate to FA metabolism that may be responsible for the lipid lowering effects of Pu-erh tea. Pu-erh tea inhibited the activity of fatty acid synthase (FASN) and acetyl-coenzyme A carboxylase (ACC), key enzymes for lipogenesis and long-chain FA synthesis, in human hepatoma HepG2 cells and rats fed a high-fructose diet and white adipose tissue of ICR mice (Way et al., 2009; Huang and Lin, 2012; Yamashita et al., 2014). Pu-erh tea extract were reported to reduced lipogenesis by down-regulating SREBP-1c and FAS in mice or SBP-1 and its target SCD in *Caenorhaditis elegans* to suppress the fat accumulation (Shimamura et al., 2013; Ding et al., 2015). Our results further confirmed the results that Pu-erh tea inhibited expression of SREBP1c, CHREBP, ACC-1 and FAS. Moreover, Pu-erh tea promoted expression of PPARα, CPT1α, and ACOX-1 to enhance the β-oxidation of FA in the liver.

Taken together, the results generated from our study suggest that Pu-erh tea is effective in improving the impaired glucose homeostasis and insulin sensitivity through altering the FA metabolic pathways. These results may provide new insights in the anti-obese and lipid-lowering effects of Pu-erh tea and perhaps new therapeutic or interventional approaches for treating obesity and diabetes. However, further investigations of Pu-erh tea on human subjects with obesity or diabetes are warranted to confirm these outcomes identified in mouse models.

## Author Contributions

WJ conceptualized the study and designed the research. FH performed the experiments and the overall analysis with the help from AZ and XZ. YZ, SL, SW, KG, CQ, and QZ contributed to the animal experiments. SW, SL, and CY contributed to data analysis. FH and WJ wrote the manuscript with the input of other co-authors.

## Conflict of Interest Statement

The authors declare that the research was conducted in the absence of any commercial or financial relationships that could be construed as a potential conflict of interest.

## Abbreviations

ACC-1: acetyl-CoA carboxylase-1
ACOX-1: acy-coenzyme A oxidase-1
C10:0: *n*-decanoic acid
C12:0: laurostearic acid
C12:1 *cis*-11: *cis*-11-dodecenoic acid
C13:0: tridecanoic acid
C14:0 iso: isomyristic acid
C14:0: myristic acid
C14:1 n5: *cis*-9-tetradecenoic acid
C15:0: pentadecanoic acid
C15:1 n5: *cis*-10-pentadecenoic acid
C16:0 iso: isopalmitic acid
C16:0, PA: palmitic acid
C16:1 n7: palmitoleic acid
C16:2: 9Z,12Z-hexadecadienoic acid
C17:0 iso: 15-methylpalmitic acid
C17:0: heptadecanoic acid
C17:1 n7: *cis*-10-heptadecenoic acid
C18:0 iso: isostearic acid
C18:0: SA, stearic acid
C18:1 n9: oleic acid
C18:2 n6: linoleic acid
C18:3 n3: alpha-linolenic acid
C18:3 n6: gamma-linolenic acid
C19:0: nonadecanoic acid
C19:1 n9: *cis*-10-nonadecenoic acid
C19:2 n6: *cis*-10,13-nonadecadienoic acid
C20:0: eicosanoic acid
C20:1 n9: eicosenoic acid
C20:2 n6: eicosadienoic acid
C20:3 n6: DGLA, dihomogamma linolenic acid
C20:3 n9: *cis*-5,8,11-eicosatrienoic acid
C20:4 n6: AA, arachidonic acid
C20:5 n3: eicosapentaenoic acid
C21:0: heneicosanoic acid
C21:1 n9: *cis*-12-heneicosenoic acid
C22:0: behenic acid
C22:1 n9: erucic acid
C22:2 n6: *cis*-13,16-docosadienoic acid
C22:3 n3: *cis*-13,16,19-docosatrienoic acid
C22:4 n6: adrenic acid
C22:5 n3: *cis*-7,10,13,16,19-docosapentaenoic acid
C22:5 n6: *cis*-4,7,10,13,16-docosapentaenoic acid
C22:6 n3: cervonic acid
C23:0: tricosanoic acid
C23:1 n9: *cis*-14-tricosenoic acid
C24:0: lignoceric acid
C24:1 n9c: nervonic acid
C8:0: *n*-octanoic acid
C9:0: pelargonic acid
CHREBP: carbohydrate-responsive element-binding protein
CPT1α: carnitine palmitoyltransferase 1 α
FAS: fatty acid synthase
FATP2/5: fatty acid transport protein 2/5
FFA: free fatty acid
ITT: insulin tolerance test
OGTT: oral glucose tolerance test
PPARα: peroxisome proliferator-activated receptor alpha
SCD-1: stearoyl-CoA desaturase-1
SREBP1c: sterol regulatory element-binding protein 1c
