# Supplementary material for: Pu-erh Tea Regulates Fatty Acid Metabolism in Mice Under High-Fat Diet
Source: Front Pharmacol. 2019 Feb 5;10:63. doi: 10.3389/fphar.2019.00063 (PMC6370627; doi:10.3389/fphar.2019.00063)
Supplement: Supplementary file 1 [file Data_Sheet_1.docx]

SUPPLEMENTAL INFORMATION

**Supplementary Figures**


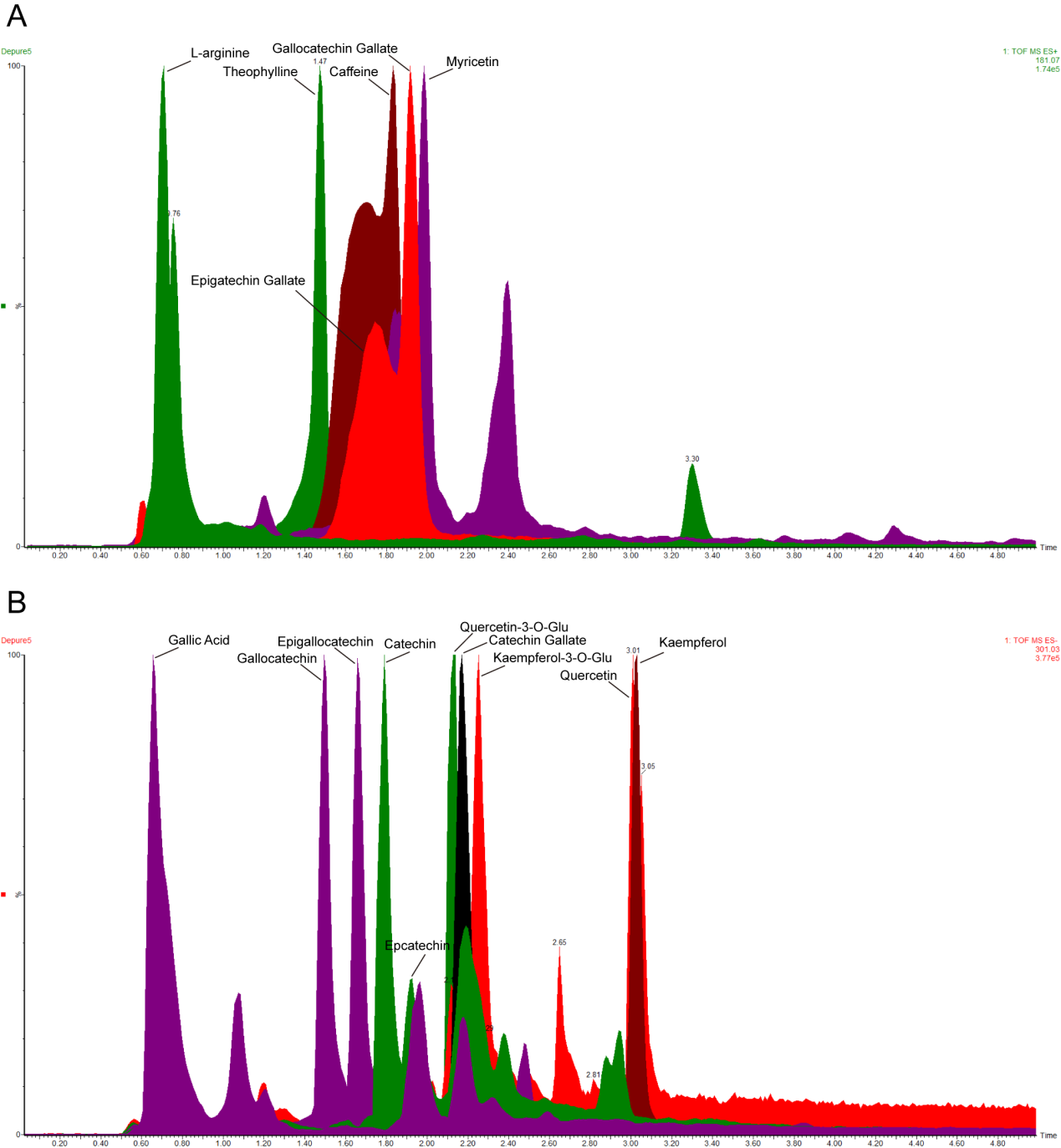


**Supplementary Figure 1. Chromatograms of the characterization of the dominating compounds in Pu-erh tea.**

The main compounds in Pu-erh tea were characterized by UPLC-QTOF-MS, among which L-arginine, Epigatechin gallate, Gallocatechin gallate, Myricetin, Caffeine and Theophylline were analyzed in positive ion mode (A) while Gallic acid, Catechin, Epcatechin, Gallocatechin, Epigallocatechin, Catechin gallate, Kaempferol, Quercetin, Quercetin-3-O-Glu and Kaempferol-3-O-Glu were anayzed in negative ion mode (B).

**Supplementary Tables**

Supplementary Table 1. Concentration of the dominating compounds in instant Pu-erh tea.

| Compound | Concentration (μmol/mg) | Ion mode |
| --- | --- | --- |
| L-arginine | 2.510207 ± 0.033507 | Positive |
| Epigatechin Gallate (EGCG) | 2.994296 ± 0.027735 | Positive |
| Gallocatechin Gallate (GCG) | 2.943378 ± 0.039116 | Positive |
| Myricetin | 0.092035 ± 0.001058 | Positive |
| Caffeine | 1.420139 ± 0.020722 | Positive |
| Theophylline | 0.270499 ± 0.008184 | Positive |
| Gallic Acid (GA) | 41.471574 ± 2.673832 | Negative |
| Catechin (C) | 2.127858 ± 0.067814 | Negative |
| Epcatechin (EC) | 0.778912 ± 0.025720 | Negative |
| Gallocatechin (GC) | 7.021337 ± 0.389047 | Negative |
| Epigallocatechin (EGC) | 5.983935 ± 0.275861 | Negative |
| Catechin Gallate (CG) | 5.691185 ± 0.152351 | Negative |
| Kaempferol | 0.083712 ± 0.004247 | Negative |
| Quercetin | 0.442899 ± 0.029055 | Negative |
| Quercetin-3-O-Glu | 0.638043 ± 0.019950 | Negative |
| Kaempferol-3-O-Glu | 0.072410 ± 0.003209 | Negative |

Supplementary Table 2. The P-value, Folder change and VIP value calculated in identification of differential FFA in serum or liver.

| FFA | Serum | | | | | | Liver | | | | | |
| --- | --- | --- | --- | --- | --- | --- | --- | --- | --- | --- | --- | --- |
|  | P value  (Mann-whitney U test) | | Folder change | | VIP (OPLSDA) | | P value  (Mann-whitney U test) | | Folder change | | VIP (OPLSDA) | |
|  | N vs H | H vs HT | N vs H | H vs HT | N vs H | H vs HT | N vs H | H vs HT | N vs H | H vs HT | N vs H | H vs HT |
| C8:0 | 0.160528 | 0.104895 | 1.771167 | 0.622615 | 0.653062 | 0.652133 | 0.002953 | 0.000155 | 0.525262 | 2.095351 | 1.03272 | 1.63315 |
| C9:0 | 0.798446 | 0.645377 | 1.025198 | 0.955093 | 0.180843 | 0.455467 | 0.573737 | 0.95913 | 0.86973 | 0.983114 | 0.308298 | 0.055478 |
| C10:0 | 0.002953 | 0.878477 | 1.360901 | 0.959085 | 0.964564 | 0.201582 | 0.278632 | 0.645377 | 0.987735 | 0.986942 | 0.034064 | 0.07583 |
| C12:0 | 0.000155 | 0.328205 | 16.80736 | 1.13913 | 1.47557 | 0.501658 | 0.104895 | 0.645377 | 0.637471 | 1.948562 | 0.256758 | 0.722427 |
| C12:1 cis:11 | 0.000311 | 0.104895 | 4.559945 | 0.675797 | 1.25823 | 0.793866 | 0.441803 | 0.505361 | 0.720875 | 1.179942 | 0.560376 | 0.49314 |
| C13:0 | 0.720901 | 0.082984 | 0.719908 | 2.206918 | 0.283642 | 0.84881 | 0.049883 | 0.95913 | 0.785005 | 0.985715 | 0.716443 | 0.078855 |
| C14:1 cis-9 | 0.000155 | 0.328205 | 3.358379 | 1.084422 | 1.27721 | 0.265632 | 0.328205 | 0.505361 | 0.785303 | 1.528134 | 0.261879 | 0.67672 |
| C14:1 trans-9 | 0.001088 | 0.234499 | 0.662266 | 1.160173 | 1.10542 | 0.447765 | 0.000155 | 0.002953 | 0.173352 | 2.696567 | 1.4833 | 1.4212 |
| C14:0 | 0.000155 | 0.160528 | 4.312685 | 1.19027 | 1.405 | 0.611265 | 0.234499 | 0.278632 | 1.076021 | 1.42454 | 0.060858 | 0.612569 |
| C14:0 iso | 0.000155 | 0.001865 | 0.332596 | 1.976892 | 1.19751 | 1.2474 | 0.002953 | 0.328205 | 0.49963 | 1.160136 | 1.16989 | 0.378587 |
| C15:0 | 0.001865 | 0.010412 | 0.735275 | 1.345211 | 1.09831 | 1.13993 | 0.000155 | 0.014763 | 0.409014 | 1.598303 | 1.47833 | 1.25272 |
| C15:0 (12-Methy) | 0.000622 | 0.000622 | 0.569715 | 1.728488 | 1.23856 | 1.38733 | 0.798446 | 0.505361 | 1.066594 | 0.895251 | 0.167535 | 0.337494 |
| C15:1 cis:10 | 0.505361 | 0.064957 | 0.847819 | 1.267423 | 0.46738 | 0.778496 | 0.000155 | 0.194872 | 0.449927 | 1.296579 | 1.43983 | 0.677891 |
| C16:2 cis-9,12 | 0.000155 | 0.004662 | 0.298605 | 1.599574 | 1.35483 | 1.25968 | 0.000155 | 0.000622 | 0.142586 | 2.459699 | 1.35186 | 1.39554 |
| C16:1 cis:9 | 0.010412 | 0.000155 | 1.469139 | 0.472809 | 0.658852 | 1.5287 | 0.000155 | 0.000155 | 1.976186 | 0.434457 | 1.40021 | 1.72736 |
| C16:1 trans:9 | 0.002953 | 0.049883 | 1.372264 | 0.815757 | 1.05103 | 0.90535 | 0.328205 | 0.720901 | 1.243725 | 0.938165 | 0.621503 | 0.228207 |
| C16:0 | 0.028127 | 0.000155 | 1.263325 | 0.689851 | 0.631087 | 1.44138 | 0.278632 | 0.004662 | 1.195284 | 0.630467 | 0.434537 | 1.24199 |
| C16:0 iso | 0.014763 | 0.010412 | 0.664412 | 1.517483 | 0.95258 | 1.1915 | 0.000155 | 0.002953 | 0.256784 | 2.120676 | 1.51326 | 1.37734 |
| C17:1 cis-10 | 0.004662 | 0.104895 | 0.733187 | 1.173085 | 0.961798 | 0.754996 | 0.001865 | 0.382284 | 0.609738 | 1.130815 | 1.23793 | 0.493417 |
| C17:0 | 0.010412 | 0.020668 | 0.70714 | 1.156316 | 0.818597 | 0.396931 | 0.000155 | 0.000622 | 0.242054 | 1.826421 | 1.47364 | 1.6388 |
| C17:0 iso | 0.000311 | 0.014763 | 0.629688 | 1.425765 | 1.26394 | 1.2105 | 0.000155 | 0.001865 | 0.278257 | 1.797655 | 1.47327 | 1.55811 |
| C17:0 (14-Methyl) | 0.000155 | 0.004662 | 0.540749 | 1.457469 | 1.34107 | 1.25656 | 0.064957 | 0.441803 | 0.567211 | 0.580323 | 0.769663 | 0.615106 |
| C18:3 cis-9,12,15 | 0.000155 | 0.001865 | 0.26255 | 1.767679 | 1.44668 | 1.31022 | 0.000155 | 0.000311 | 0.068021 | 5.689846 | 1.40479 | 1.57033 |
| C18:3 cis-6,9,12 | 0.000155 | 0.000155 | 0.268554 | 2.484375 | 1.13904 | 1.50074 | 0.037918 | 0.720901 | 0.462294 | 1.094091 | 0.849859 | 0.166763 |
| C18:2 cis-9,12 | 0.000155 | 0.000311 | 0.457759 | 1.436923 | 1.13003 | 1.40403 | 0.130381 | 0.720901 | 0.672582 | 0.861329 | 0.706092 | 0.361473 |
| C18:1 cis-9 | 0.441803 | 0.573737 | 0.861712 | 1.043766 | 0.457339 | 0.239163 | 0.234499 | 0.028127 | 1.165077 | 0.668038 | 0.615539 | 1.28427 |
| C18:1 trans-9 | 0.130381 | 0.002953 | 1.241328 | 0.70966 | 0.762691 | 1.30447 | 0.004662 | 0.720901 | 0.675783 | 1.03613 | 1.12441 | 0.161057 |
| C18:0 | 0.194872 | 0.014763 | 0.700423 | 1.31669 | 0.769199 | 0.954605 | 0.194872 | 0.234499 | 0.834581 | 0.852676 | 0.684739 | 0.871076 |
| C18:0 iso | 0.000622 | 0.000311 | 0.535742 | 1.744373 | 1.16533 | 1.49289 | 0.000155 | 0.000622 | 0.201241 | 1.8179 | 1.44176 | 1.55425 |
| C19:2 cis-10,13 | 0.000155 | 1 | 0.602307 | 1.010116 | 1.33562 | 0.05143 | 0.001865 | 0.278632 | 0.495052 | 1.113861 | 1.23341 | 0.537044 |
| C19:1 cis-10 | 0.082984 | 0.441803 | 0.692266 | 1.162972 | 0.728235 | 0.420956 | 0.000311 | 0.194872 | 0.38115 | 1.188427 | 1.33935 | 0.547399 |
| C19:1 trans-10 | 0.234499 | 0.006993 | 0.798935 | 1.52622 | 0.541527 | 1.1167 | 0.000311 | 0.049883 | 0.320038 | 1.52711 | 1.42065 | 1.07352 |
| C19:0 | 0.000622 | 0.001088 | 0.633885 | 1.747577 | 1.22173 | 1.34234 | 0.000155 | 0.001088 | 0.148296 | 1.896354 | 1.38814 | 1.55232 |
| C20:5 cis-5,8,11,14,17 | 0.001865 | 0.000155 | 0.476122 | 1.815352 | 1.03412 | 1.37442 | 0.010412 | 0.441803 | 0.352625 | 1.347535 | 0.869484 | 0.569194 |
| C20:4 cis-5,8,11,14 | 0.014763 | 0.441803 | 0.659315 | 1.096443 | 0.981208 | 0.455617 | 0.064957 | 0.194872 | 0.811304 | 0.784646 | 0.546886 | 0.616059 |
| C20:3 cis-8,11,14 | 0.049883 | 0.001865 | 1.290488 | 0.62148 | 0.861884 | 1.45459 | 0.004662 | 0.000155 | 1.465187 | 0.461656 | 1.11186 | 1.69581 |
| C20:3 cis-5,8,11 | 0.000622 | 0.004662 | 1.623798 | 0.710082 | 1.13542 | 1.09084 | 0.004662 | 0.328205 | 1.939833 | 0.792757 | 1.11614 | 0.617494 |
| C20:2 cis-11,14 | 0.004662 | 0.382284 | 0.636681 | 0.922615 | 0.880211 | 0.417829 | 0.720901 | 0.037918 | 1.21532 | 0.503464 | 0.33876 | 1.13348 |
| C20:1 cis-11 | 0.004662 | 0.082984 | 0.676664 | 1.23689 | 1.0612 | 0.616172 | 0.505361 | 0.064957 | 1.24194 | 0.660875 | 0.529721 | 1.02122 |
| C20:0 | 0.878477 | 0.95913 | 0.949908 | 1.083318 | 0.223541 | 0.245975 | 0.505361 | 0.082984 | 1.091427 | 0.786883 | 0.345262 | 0.996223 |
| C21:0 | 0.382284 | 0.049883 | 0.712552 | 2.12198 | 0.554853 | 0.794179 | 0.000622 | 0.95913 | 0.643765 | 0.958906 | 1.22063 | 0.251496 |
| C21:1 cis-12 | 0.028127 | 0.020668 | 0.692077 | 1.828505 | 0.923711 | 0.859566 | 0.020668 | 0.194872 | 0.694536 | 0.791504 | 0.933154 | 0.802123 |
| C22:6 cis-4,7,10,13,16,19 | 0.001865 | 0.000155 | 0.518135 | 1.629453 | 1.07953 | 1.45388 | 0.000155 | 0.002953 | 0.413491 | 1.480756 | 1.3724 | 1.4161 |
| C22:5 cis-7,10,13,16,19 | 0.278632 | 0.020668 | 0.710265 | 1.377464 | 0.703859 | 1.12207 | 0.082984 | 0.878477 | 0.69095 | 0.906649 | 0.754761 | 0.316329 |
| C22:5 cis-4,7,10,13,16 | 0.130381 | 0.194872 | 1.243325 | 0.850679 | 0.715179 | 0.551086 | 0.573737 | 0.505361 | 1.062448 | 0.864842 | 0.188678 | 0.424707 |
| C22:4 cis-7,10,13,16 | 0.014763 | 0.328205 | 0.826862 | 0.903526 | 0.828855 | 0.406597 | 0.104895 | 0.645377 | 0.786061 | 1.07657 | 0.791909 | 0.342299 |
| C22:3 cis-13,16,19 | 0.001088 | 0.004662 | 1.309266 | 0.655871 | 1.14969 | 1.41402 | 0.000155 | 0.004662 | 1.792813 | 0.626485 | 1.27963 | 1.32411 |
| C22:2 cis-13,16 | 0.014763 | 0.645377 | 0.83164 | 0.99343 | 0.997149 | 0.041209 | 0.028127 | 0.006993 | 1.419589 | 0.630468 | 0.939419 | 1.29221 |
| C22:1 cis-13 | 0.104895 | 0.328205 | 0.759049 | 1.347564 | 0.721758 | 0.636722 | 0.000311 | 0.082984 | 2.114746 | 0.710121 | 1.25908 | 0.98244 |
| C22:0 | 0.000311 | 0.001865 | 0.361589 | 2.396714 | 1.22633 | 0.99135 | 0.505361 | 0.645377 | 1.39378 | 0.67431 | 0.395942 | 0.653741 |
| C23:1 cis-14 | 0.020668 | 0.001088 | 0.56659 | 1.965763 | 0.95885 | 1.11926 | 0.000155 | 0.037918 | 0.36933 | 1.413888 | 1.42802 | 1.05825 |
| C23:0 | 0.028127 | 0.001088 | 0.453467 | 3.075322 | 0.933477 | 1.19973 | 0.573737 | 0.049883 | 1.061659 | 0.733937 | 0.181797 | 1.0935 |
| C24:1 cis-15 | 0.001088 | 0.001865 | 0.407854 | 2.221202 | 1.187 | 1.36054 | 0.382284 | 0.328205 | 0.871671 | 0.848284 | 0.491629 | 0.779859 |
| C24:0 | 0.014763 | 0.194872 | 0.514397 | 1.758554 | 0.891233 | 0.67243 | 0.328205 | 0.014763 | 1.147733 | 0.723457 | 0.382872 | 1.22528 |
